# A Novel Multi-Focus Image Fusion Network with U-Shape Structure

**DOI:** 10.3390/s20143901

**Published:** 2020-07-13

**Authors:** Tao Pan, Jiaqin Jiang, Jian Yao, Bin Wang, Bin Tan

**Affiliations:** 1 School of Remote Sensing and Information Engineering, Wuhan University, Wuhan 430000, China; pantao@whu.edu.cn (T.P.); jiangjiaqin@whu.edu.cn (J.J.); tanbin@whu.edu.cn (B.T.); 2 School of Artificial Intelligence, The Open University of Guangdong, Guangzhou 510000, China; 3 College of Electronic Information, Micro-Nano technology College, Qingdao University, Qingdao 266071, China; dzxywb@qdu.edu.cn

**Keywords:** multi-focus image fusion, U-shape network, Siamese encoder, spatial pyramid pooling, hybrid loss

## Abstract

Multi-focus image fusion has become a very practical image processing task. It uses multiple images focused on various depth planes to create an all-in-focus image. Although extensive studies have been produced, the performance of existing methods is still limited by the inaccurate detection of the focus regions for fusion. Therefore, in this paper, we proposed a novel U-shape network which can generate an accurate decision map for the multi-focus image fusion. The Siamese encoder of our U-shape network can preserve the low-level cues with rich spatial details and high-level semantic information from the source images separately. Moreover, we introduce the ResBlocks to expand the receptive field, which can enhance the ability of our network to distinguish between focus and defocus regions. Moreover, in the bridge stage between the encoder and decoder, the spatial pyramid pooling is adopted as a global perception fusion module to capture sufficient context information for the learning of the decision map. Finally, we use a hybrid loss that combines the binary cross-entropy loss and the structural similarity loss for supervision. Extensive experiments have demonstrated that the proposed method can achieve the state-of-the-art performance.

## 1. Introduction

**Figure 1:**
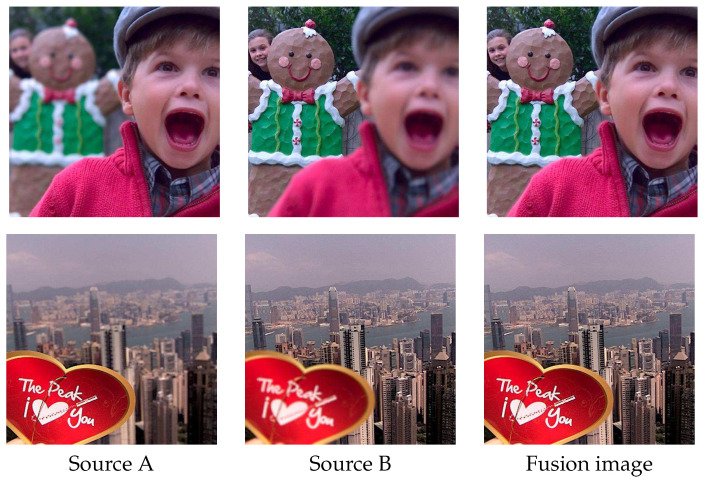
Examples of multi-focus image fusion with two source images. Source A focuses on the foreground and Source B focuses on the background. Fusion image is the fusion result obtained by our method.

Obtaining an all-in-focus image of a scene is essential for many computer vision and image analysis tasks. However, due to the limited depth of field (DoF) of the optical lens and the various depth of objects in a scene, it is difficult to capture an image where all objects are focused in one shot. Multi-focus image fusion is a common method used to solve this issue by the way of image processing. It fuses multiple images of the same scene taken with different focal parameters to create an all-in-focus image with all objects in the scene clear, as shown in Figure 1.

The existing multi-focus image fusion methods can be divided into two categories, i.e., the transform domain methods and the spatial domain methods [1,2]. The transform domain methods are usually based on the multi-scale transformation (MST) theories which generally contain three stages. First, the source images are decomposed into a special domain according to a certain transform method. Then, the transformed coefficients are fused based on artificially designed fusion criteria. Finally, the fused coefficients are transformed back to the original image domain by an inverse transform to generate the final fused image. There are many well-known methods such as laplacian pyramid (LP) [3], ratio of low-pass pyramid (RP) [4], the sparse representation (SR) [5], discrete wavelet transform (DWT) [6], dual-tree complex wavelet transform (DTCWT) [7], curvelet transform (CVT) [8], and nonsubsampled contourlet transform (NSCT) [9], etc. Due to the imperfect selections of transform domains and fusion rules, the fusion results of these algorithms are often indistinct.

Spatial domain methods can be divided into block-based methods, region-based methods and pixel-based methods. The block-based methods, such as spatial frequency [10], decompose the source image into blocks of a certain size, and then detects clear image blocks by a designed focus level measurement. Since both focus and defocus pixels may appear in one block, the performance of block-based algorithms is related to whether the block size is set appropriately. Some improved methods [11,12,13] try to solve this problem by adaptively adjusting the block size, but the fusion results still inevitably have block effects. The region-based methods [14,15] use various image segmentation algorithms to extract focus regions. The effectiveness of these methods is limited by the accuracy of the image segmentation. The pixel-based methods generate a decision map pixel-by-pixel to guide the fusion processing. Typical examples of this type of method are the guided filtering (GF) [16], the dense SIFT (DSIFT) [17] and the multi-scale weighted gradient (MWG) [18].

In the above-mentioned methods, both transform domain methods and spatial domain methods, the focus level measurements and the fusion rules are two important factors that affect the fusion quality. However, they usually need to be designed manually. Due to the complexity and diversity of real-world scenarios, it is difficult to get a perfect design that considers all factors that affect the quality of fusion.

Recently, inspired by the successful application of deep learning (DL) in image processing fields, some multi-focus image fusion methods based on convolutional neural network (CNN) have emerged. Liu et al. [19] first proposed a CNN-based multi-focus fusion scheme, which generates an initial decision map through a binary classification network, and then the precise decision map is obtained through a series of post-processing refinements for guiding fusion. Tang et al. [20] proposed a pixel-level convolutional neural network (P-CNN) to distinguish between focus and defocus pixels. Guo et al. [21] used the fully convolutional neural network for multi-focus image fusion. However, the initial decision map generated by the network still needs to be refined by the fully connected conditional random field (CRF). The initial decision maps obtained by the above CNN-based and FCN-based methods usually have a large number of blurs and errors, and are inaccurate at the boundaries of the focus and defocus regions, so they cannot be used directly to guide the fusion of multi-focus images. Therefore, the quality of the final focus decision map depends largely on various post-processing techniques, such as small region removal, edge preservation filtering, Consistency Verification (CV), CRF optimization, etc. Amin-Naji et al. [22] continued to propose a new FCN-based multi-focus image fusion network, which can provide a relatively clean initial decision map. However, it uses a patch-based strategy, which results in redundant calculations in the inference process and difficult segmentation at the boundaries of the focus and defocus regions. Besides generating decision maps for fusion, some works attempted to generate the fused image directly. Parbhakar et al. [23] proposed an unsupervised CNN-based network called DeepFuse for fusing multiple-exposed images. Zhao et al. [24] developed a multi-level deep supervised network (MLCNN) to directly fuse and enhance multi-focus images by combining multi-level features. Yan et al. [25] proposed an unsupervised CNN-based approach for fusion. However, these methods usually have some blurred effects and may have artifacts that are unrelated to the source images due to the lack of precise focus regions detection.

To solve the above problems, in this work, we propose a novel U-shape Siamese network architecture for multi-focus image fusion. The main contributions of this article can be summarized as: (1) A novel U-shape model with Siamese encoder is proposed to generate a satisfactory decision map for guiding fusion; (2) ResBlocks are introduced to expand the receptive field, which can make our network distinguish between focus and defocus regions in the source images well; (3) ResBlocks are introduced to expand the receptive field, which can make our network distinguish between focus and defocus regions in the source images well; (4) The outputs of each level in the decoder are deeply supervised by a hybrid loss function combining binary cross-entropy (BCE) loss and structural similarity (SSIM) loss.

The rest of the paper is organized as follows. Section 2 reviews related works. Section 3 describes the proposed multi-focus image fusion method in detail. Section 4 verifies the effectiveness of our proposed method through experimental results. Finally, we conclude in Section 5.

## 2. Related Work

### 2.1. Deep Learning for Multi-Focus Image Fusion

With the success of deep learning in computer vision and image processing, some recent works have applied it to the multi-focus image fusion task. The key to most DL-based methods is to accurately detect the focus regions from the source multi-focus images. Liu et al. [19] first attempted to introduce convolutional neural network (CNN) to this task. They designed a Siamese CNN and trained it with labeled focus and defocus image patches. The features of the two source images respectively extracted through the Siamese architecture are cascaded and input to the fully connected layers for binary classification. Du et al. [26] regarded the task of multi-focus image fusion as image segmentation, and obtained the coarse segmentation result by constructing a multi-scale CNN which takes image patches with different size as input, then adopted some image process techniques to refine the segmentation result. Tang et al. [20] proposed a pixel-level convolutional neural network (P-CNN), which can measure the focus levels on each pixel of the source image. It classifies each pixel of the source image into three categories: focus, defocus and the unknown to generate an initial decision map. Guo et al. [21] adopted full convolutional neural network (FCN) for multi-focus image fusion. Compared with other methods, it uses a very deep network to achieve semantic segmentation of the focus and defocus regions from the source images. However, it still uses a fully connected conditional random field as post-processing to refine the initial decision map. Recently, Guo et al. [27] attempted to build a mapping from source images to decision maps through conditional generative adversarial networks (cGAN). Farid et al. [28] used a content adaptive blurring (CAB) algorithm to distinguish the focus and defocus regions. Theoretically, the quality of the focus patches would be degraded obviously after several blurring; however, the defocus patches have little changes. According to the absolute difference of the original image and the CAB-blurred image, the initial segmentation map was obtained. Then the morphological operators and graph-cut techniques were introduced to improve the segmentation result. In the above methods, the network can only provide an undesirable initial decision map with many errors. The final satisfactory decision map can only be obtained after a series of post-processing steps for refinement, such as small region removal, guided filters, Consistency Verification (CV), CRF optimization, etc.

### 2.2. U-Shape Networks

Since U-Net [29] was first proposed by Ronneberger et al. for biomedical image segmentation, it has received widespread attention for its ability to construct rich feature maps by the top-down pathways. The key to the good performance of U-shape network in the field of semantic segmentation is that its architecture can combine low-level cues with spatial details and high-level semantic information. In addition, compared to FCN which requires a lot of memory and calculation time, U-shape network has the advantages of small memory and fast inference speed because of its simple structure. Inspired by these, in this paper, we design a U-shape structured network for multi-focus image fusion and deeply supervise its coarse-to-fine outputs at different stages of decoder.

## 3. The Proposed Method

### 3.1. Method Overview

Generally speaking, the framework of multi-focus image fusion can be summarized by the fusion process of two images. Specifically, the final fusion result of multiple images can be obtained by fusing the source images one by one in sequence. Therefore, the proposed method only takes into account the case of dual focus images.

**Figure 2:**
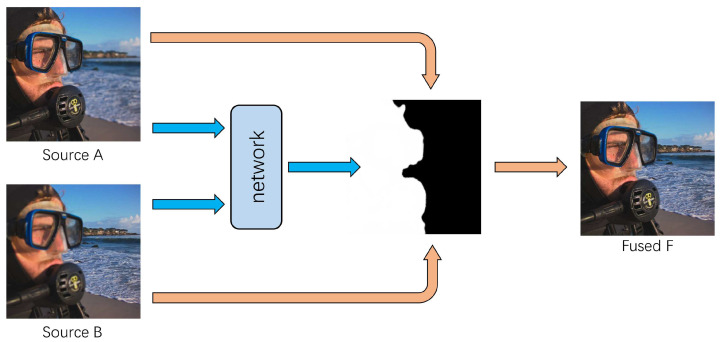
The schematic diagram of the proposed method. Source A and Source B are input into the network to get a decision map that can be used to guide image fusion.

A schematic diagram of the proposed method is shown in Figure 2. First, a synthetic method is used to generate pairs of foreground-focused and background-focused images for training. Then, the proposed network receives the pair of multi-focus images as the input and generates a high-quality decision map. Finally, in the fusion stage, without post-processing steps such as small region removal, guided filters, Consistency Verification (CV), CRF optimization, etc., the fusion results are produced by directly applying the decision map according to the following formula:

(1) $$F=I_{A}\times M+I_{B}\times \left(1-M\right),$$

where *F* is the fused image, $I_{A}$ and $I_{B}$ are the source images, and *M* is the decision map generated by the network.

### 3.2. Network Architecture

**Figure 3:**
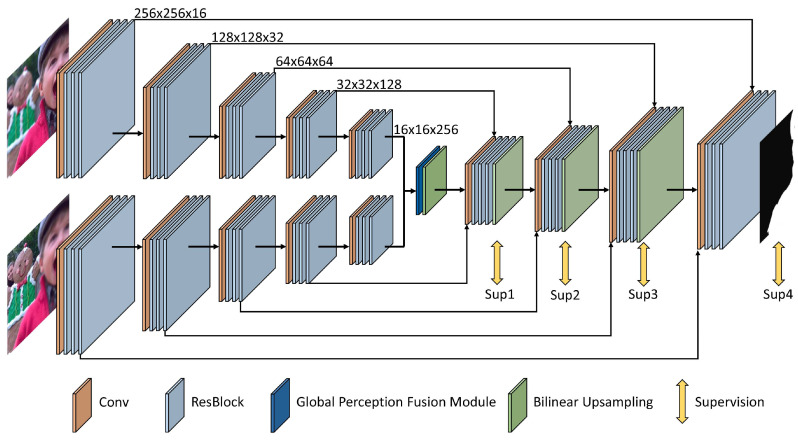
The architecture of our proposed U-shape multi-focus image fusion network with a Siamese encoder. The Siamese encoder extracts the features of two input images simultaneously, and fuses them through the global perception fusion module in the bridge stage of the network. Each stage of the decoder cascades the feature maps from the corresponding stage of the Siamese encoder and outputs a decision map, which participates in the supervision of the training. ResBlocks are added in each stage of the encoder and decoder.

The architecture of our network for multi-focus fusion is shown in Figure 3. In contrast to those existing methods which divide the source images into patch pairs and always generate inaccurate decision maps by classifying the clear and unclear patches, our proposed networks accepts two source images as the inputs and directly outputs a decision map with the full resolution. By this way, we can obtain a more accurate decision map especially in the boundaries of the focus and defocus regions. In addition, since the proposed network can directly consume the whole image instead of the image patches, the computation source can be effectively reduced, and the entire fusion process is efficient.

To accurately generate a high-quality decision map for multi-focus image fusion, we propose three improvements based on the U-shape network backbone, which are (1) using the Siamese encoder in our U-shape network to retain the multi-level features from two source images, (2) introducing ResBlocks to better perceive the focus characteristics of the images, and (3) adding a global perception fusion module to capture context information. We will explain them in detail.

#### 3.2.1. U-Shape Siamese Network

Inspired by the success of U-Net [29] in semantic segmentation and other fields, we apply the similar symmetrical U-shape structure as the backbone of our network. The input images are fed into the encoder to generate the low-level features with spatial details in the high-resolution and the high-level features with semantic information in the low resolution. Then, the skip connections are used to transmit the low-level features with rich details from the encoder to the decoder. By this way, we can make good use of the features from the encoder with both semantic information and spatial details. Moreover, the skip connections can improve the propagation of the gradient information and thus speed up the convergence during training.

It is worth noticing that our network takes two images as the inputs, so the typical single-branch encoder–decoder structure needs to be modified for our multi-focus fusion task. Therefore, we adopt the Siamese network as the encoder of our network, which consists of two weight-shared encoder branches to process the input images, respectively. Then, the output features of the Siamese network are further fused through the global perception fusion module which will be described in Section 3.2.3 and fed into the decoder. Compared with feeding the combined input images to a single-branch network structure, our network with the Siamese encoder is more interpretable as it forces to perceive the focus characteristics of two source images in the same way. Moreover, the Siamese encoder structure can well preserve the multi-level features of the input images separately, which can be further fused in the decoder to benefit the learning of the decision maps. To improve the learning process, we produce the decision maps both in the final stage and the medial stages of the decoder. In particular, a 7×7 convolution layer followed by a bilinear upsampling operation and a sigmoid operation is applied to the output of each stage of the decoder to generate the decision maps during training. The decision maps with the same spatial resolution as ground truths are all involved in the loss calculation as described in Section 3.3.

#### 3.2.2. Encoder/Decoder ResBlocks

In the typical structure of the U-Net, several flat convolutions are used in each stage. However, for the task of decision map generation, such structure is not enough to generate a good result. This is because that the network needs to learn to measure the focus levels of the images which cannot be well carried out by a small receptive filed. One solution is to stack more stages in the encoder. However, too many stages will make the spatial size of the encoder features too small to retain enough geometric information for the decoder to recover the high-resolution feature maps. Another solution is to use more flat convolutions in each stage to enlarge the receptive field. However, this scheme will increase the depth of the network and make the convergence difficult. Thus, in this paper, we borrow the idea of the ResNet [30] and form a deep network with large receptive field by replacing the flat convolution with more ResBlocks at each stages of the encoder and the decoder. As described in the ResNet [30], the ResBlock can improve the gradient propagation and enable the training of deep networks. Specifically, three ResBlocks are introduced at each stage of the encoder and decoder as shown in Figure 3.

#### 3.2.3. Global Perception Fusion Module

**Figure 4:**
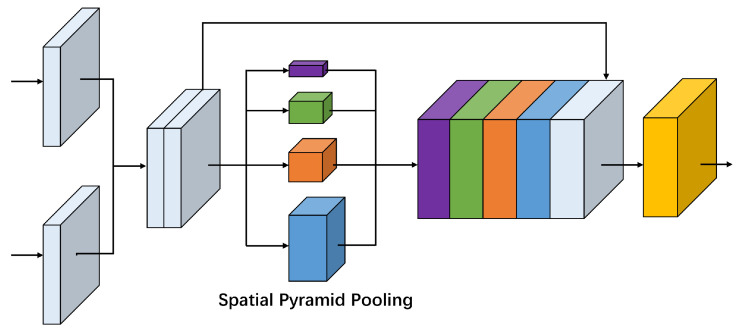
The proposed global perception fusion module based on spatial pyramid pooling. When the features from the two branches of the Siamese encoder are fused, four scale (1 × 1, 2 × 2, 3 × 3, 6 × 6) pooling operations follow. Then these feature maps are upsampled to the same spatial resolution and cascaded together.

It is a big challenge to distinguish the focus or defocus regions in the homogeneous area, because the texture is usually lacking in these places so that there is almost no difference in appearance whether be focus or defocus. In addition, the presence of focus or defocus objects with different sizes in a real scene also requires the network to be invariant to different object scales. Therefore, to solve these problems, we adopt the spatial pyramid pooling as used in [31] to serve as a global perception fusion module (GPFM) in the bridge stage of our network, as shown in Figure 4. In this way, when the features from the two branches of the Siamese encoder are fused, the global prior constraint and multiple scale information can be preserved simultaneously, which helps to get reliable classification results for homogeneous regions and multi-scale objects. Specifically, the two feature maps with 256 channels from the Siamese encoder will be cascaded into a feature map with 512 channels and pooled into 4 scales: 1×1, 2×2, 3×3, 6×6. Then we upsample them to the same spatial resolution and finally cascade them together.

### 3.3. Loss Function

The proposed network can get different resolution outputs at the decoder stages, so the model can be supervised by them together. Our training goals are the summation of losses at all stages:

(2) $$L=\sum_{k=1}^{K}w_{k}\times l^{k},$$

where $l^{k}$ is the loss of the *k*-th stage output, $w_{k}$ is the weight of the loss, and *K* is the number of outputs. Here $w_{k}$ is all set to 1 and *K* is 4 according to our network.

The loss $l^{k}$ of each stage is a hybrid loss function containing two parts. It can be defined as:

(3) $$l^{k}=l_{BCE}^{k}+l_{SSIM}^{k},$$

where $l_{BCE}^{k}$ is the BCE loss and $l_{SSIM}^{k}$ is the SSIM loss.

BCE [32] loss is a binary cross-entropy loss function, which is commonly used in image classification and segmentation tasks. It can be calculated using the following formula:

(4) $$l_{BCE}=-\sum_{i=1,j=1}^{M,N}\left(G_{i,j}\log M_{i,j}+\left(1-G_{i,j}\right)\log\left(1-M_{i,j}\right)\right),$$

where $G_{i,j}$ is the ground truth label at pixel (i,j) and $M_{i,j}$ is the predicted probability value of the output decision map at pixel (i,j).

SSIM [33] is used for image quality assessment from the perspective of the human visual system. It can capture spatial structure information in an image. Therefore, we add SSIM loss to the objective to enhance the structural constraints of the decision map.

The SSIM loss of two images *x* and *y* is defined as:

(5) $$l_{SSIM}=1-\frac{\left(2\mu_{x}\mu_{y}+C_{1}\right)\left(2\sigma_{xy}+C_{2}\right)}{\left(\mu_{x}^{2}+\mu_{y}^{2}+C_{1}\right)\left(\sigma_{x}^{2}+\sigma_{y}^{2}+C_{2}\right)},$$

where $\mu_{x}$ and $\mu_{y}$ are the mean of *x* and *y*, $\sigma_{x}$ and $\sigma_{x}$ denote the standard deviations of *x* and *y*, and $\sigma_{xy}$ is their covariance. $C_{1}$ and $C_{2}$ are two small constants used to avoid dividing by zero.

## 4. Experiments and Analysis

### 4.1. Data Preparation

It is well known that supervised deep learning methods require large amounts of labeled training data. For our proposed network that generates a decision map to guide the fusion process, a large amount of multi-focus image pairs with binary decision maps are needed. However, the publicly available Lytro [34] dataset has only 20 pairs of multi-focus images, without corresponding all-in-focus images and binary decision maps. Therefore, we use a synthetic method to generate a sufficient number of multi-focus images as our training dataset.

We adopt the method introduced by Guo et al. [27] to synthesize 5092 pairs of multi-focus image pairs on the PASCAL VOC 2012 image dataset [35]. We select 4092 pairs as the training set and 1000 pairs as the validation set to select the optimal model. Please note that images in the validation set are not included in the training set. This method of synthesizing multi-focus image pairs uses a normalized disk point spread function (PSF) to simulate blur and the PSF $H_{x,y}$ is formulated as

(6) $$H_{x,y}=\left\{\begin{array}{c} \frac{1}{\pi R^{2}},\sqrt{x^{2}+y^{2}}\leq R\\ 0,\sqrt{x^{2}+y^{2}}>R, \end{array}\right.$$

where *x* and *y* are coordinate indexes and *R* denotes the disk radius. Different levels of blur can be simulated by PSF with different disk radius.

### 4.2. Experimental Setup

**Figure 5:**
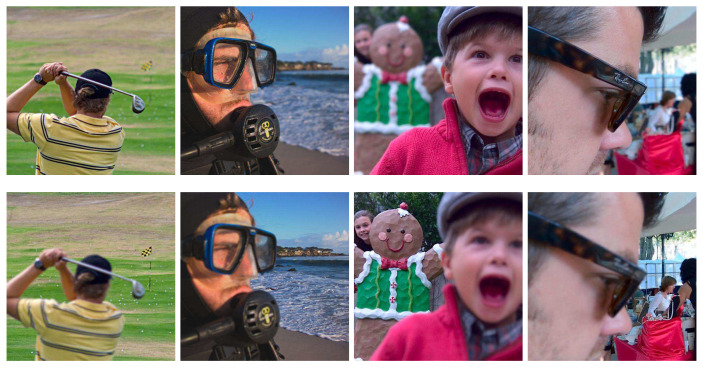
Examples of the source image pairs from the Lytro color multi-focus dataset.

To verify the effectiveness of the proposed method, we selected a public dataset Lytro [34] with 20 pairs of multi-focus images as the test data. The size of the images is 520×520, and some of which are showed in Figure 5.

In the following experiments on Lytro dataset, we compare the proposed method with many well-known multi-focus image fusion methods, including the Laplacian pyramid (LP) [3], the ratio of low-pass pyramid (RP) [4], the nonsubsampled contourlet transform (NSCT) [9], the discrete wavelet transform (DWT) [6], dual-tree complex wavelet transform (DTCWT) [7], the sparse representation (SR) [5], the curvelet transform (CVT) [8], the multi-scale weighted gradient (MWG) [18], the dense SIFT (DSIFT) [17], the DeepFuse [23] and the CNN-based [19]. In addition, the parameters of these methods are set to the recommended values consistent with their original papers.

### 4.3. Implementation

During the training phase, each pair of training images are resized to 256×256. We use Adam [36] optimizer to train our network with parameters $\beta_{1}$ = 0.9, $\beta_{2}$ = 0.999, eps = 1 × 10${}^{-8}$ and weight decay = 0. The initial learning rate is 1 × 10${}^{-4}$ and it is decreased by 0.8 times every two epochs. The weights in all layers are initialized by uniform distribution function. The batch size is set to 8 and the total epochs are set to 50. During the test phase, the input images are resized to 256×256 and then fed into the network. As the size of the output decision map is also 256×256, it will be resized back to the spatial resolution of the original input images. Please note that we use bilinear interpolation for resizing.

We implemented our network using the public deep learning framework PyTorch [37]. A GTX 1080ti GPU with 11GB of memory is used for training and testing.

### 4.4. Quantitative Evaluation Metrics

As we all know, due to the lack of corresponding all-in-focus images as ground truth, it is difficult to quantitatively evaluate the quality of multi-focus image fusion. Therefore, to comprehensively evaluate the performance of the fusion algorithms, multiple different evaluation indicators should be used. Commonly used fusion metrics can be divided into four categories: information theory-based, image feature-based, image structure similarity-based and human perception-based metrics. We adopt 4 kinds of metrics, covering the above 4 categories, namely $Q_{NMI}$, $Q^{AB/F}$, $Q_{Y}$, $Q_{CB}$. Please note that for these metrics, higher values indicate better fusion quality. Next we introduce these metrics in detail.

#### 4.4.1. Normalized Mutual Information $Q_{NMI}$

Mutual information is a metric based on information theory, which indicates how much information in the original image is contained in the fused image. The normalized mutual information [38] overcomes the instability of the traditional one. The definition of $Q_{NMI}$ is as follows:

(7) $$Q_{NMI}=2\times \left(\frac{MI_{AF}}{H_{A}+H_{F}}+\frac{MI_{BF}}{H_{B}+H_{F}}\right),$$

where $MI_{AF}$ and $MI_{BF}$ are the mutual information between images *A* and *F*, *B* and *F* respectively; $H_{A}$, $H_{B}$ and $H_{F}$ are the entropy of images *A*, *B* and *F* respectively.

#### 4.4.2. Gradient-Based Fusion Metric $Q^{AB/F}$

$Q^{AB/F}$ [39] is an image gradient-based metric used to evaluate the edge information retained from the source image to the fused image. It is defined as follows:

(8) $$\begin{array}{cc} \; & Q^{AB/F}=\frac{\sum_{m=1}^{M}\;\sum_{n=1}^{N}\left(Q^{AF}\left(m\;,\;n\right)w^{A}\left(m\;,\;n\right)\;+\;Q^{BF}\left(m\;,\;n\right)w^{B}\left(m\;,\;n\right)\right)}{\sum_{m=1}^{M}\;\sum_{n=1}^{N}\left(w^{A}\left(m\;,\;n\right)\;+\;w^{B}\left(m\;,\;n\right)\right)}, \end{array}$$

where

(9) $$Q^{AF}\left(x,y\right)=Q_{g}^{AF}\left(x,y\right)Q_{\alpha }^{AF}\left(x,y\right),$$

$Q_{g}^{AF}\left(x,y\right)$ and $Q_{\alpha }^{AF}\left(x,y\right)$ represent the edge strength and orientation preservation values at coordinates (x,y), respectively. The calculation of $Q^{BF}$ is similar to $Q^{AF}$. $w^{A}\left(m,n\right)$ and $w^{B}\left(m,n\right)$ are the weight coefficients of the source image *A* and *B*.

#### 4.4.3. Yang’s Metric $Q_{Y}$

The structural similarity-based metric $Q_{Y}$ [40] can evaluate how much image structure information from the source image is retained in the fused image. Its formula is as follows:

(10) $$Q_{Y}=\left\{\begin{array}{c} \lambda (\omega )SSIM(A,F|\omega )\;+\;(1\;-\;\lambda (\omega ))SSIM(B,F|\omega ),SSIM(A,F|\omega )\geq 0.75,\\ \max(SSIM(A,F|\omega ),SSIM(B,F|\omega )),SSIM(A,B|\omega )<0.75. \end{array}\right.$$

where SSIM is the image structure similarity, ω is the local window, λ(ω) and (1−λ(ω)) are the weights of two source images under the local window:

(11) $$\lambda \left(\omega \right)=\frac{s(A|\omega )}{s(A|\omega )+s(B|\omega )},$$

where s(A|ω) and s(B|ω) are the variances of the source images *A* and *B* on the local window ω.

#### 4.4.4. Chen-Blum Metric $Q_{CB}$

The human perception-based metric mainly uses human visual features to measure the fused image. The formula of $Q_{CB}$ [41] is:

(12) $$Q_{CB}=\bar{Q_{GQM}},$$

$Q_{GQM}$ is a global quality map, which is calculated as follows:

(13) $$Q_{GQM}\left(x,y\right)=\lambda_{A}\left(x,y\right)Q_{AF}\left(x,y\right)+\lambda_{B}\left(x,y\right)Q_{BF}\left(x,y\right),$$

where $Q_{AF}$ and $Q_{BF}$ are the contrasts retained in the fused image *F* from the source image *A* and the source image *B*, respectively. $\lambda_{A}$ and $\lambda_{B}$ are saliency maps for $Q_{AF}$ and $Q_{BF}$, respectively.

### 4.5. Visual Results

**Figure 6:**
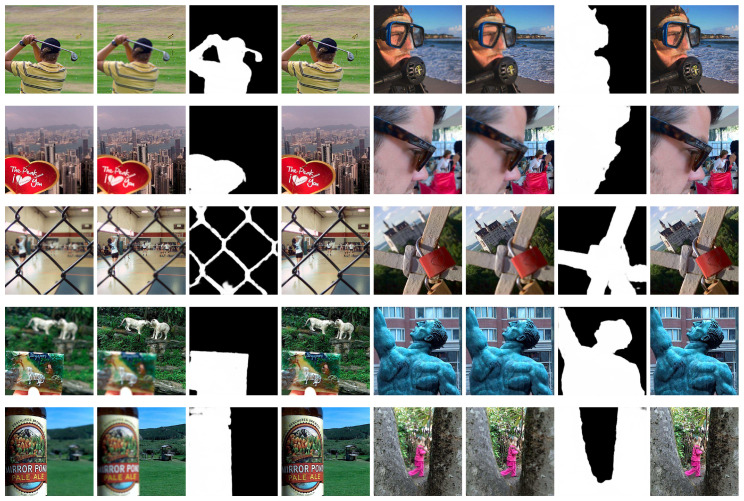
Visualization results of our method on Lytro dataset. Columns 1, 2, 5, 6 show the source multi-focus image pairs, columns 3, 7 show the decision map generated by the proposed network, and columns 4, 8 are the corresponding fusion results.

As shown in Figure 6, we visualize a part of the decision maps generated by our network and the final fusion results. Please note that these decision maps do not go through any post-processing steps, such as consistency verification (CV), small region removal, morphological operations, guided filters and conditional random field optimization (CRF). In addition, we directly use the decision maps generated by the proposed network to guide the fusion process. As we can see, the decision maps are clean enough without obvious misclassification regions, and the boundaries between focus and defocus regions are clear. It means that the designed network can completely and clearly detect the focus regions from the source images.

**Figure 7:**
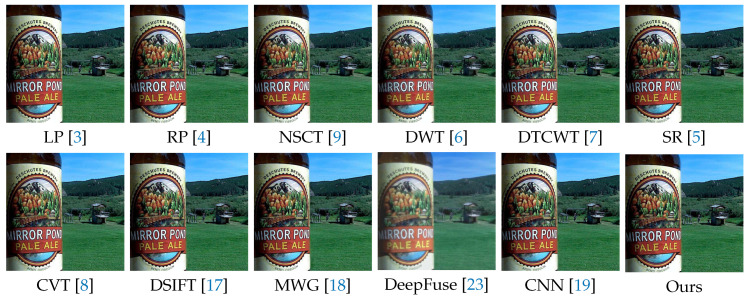
The fusion results of ’Lytro-09’ using various methods.

**Figure 8:**
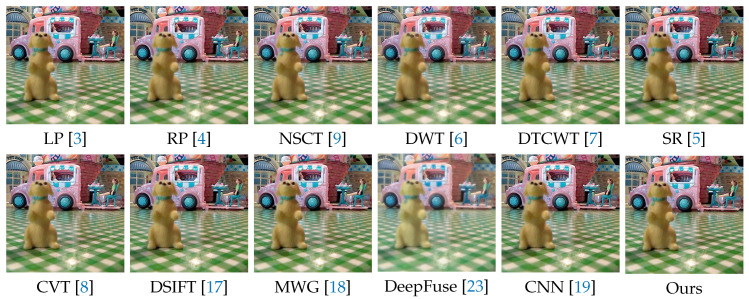
The fusion results of ’Lytro-17’ using various methods.

**Figure 9:**
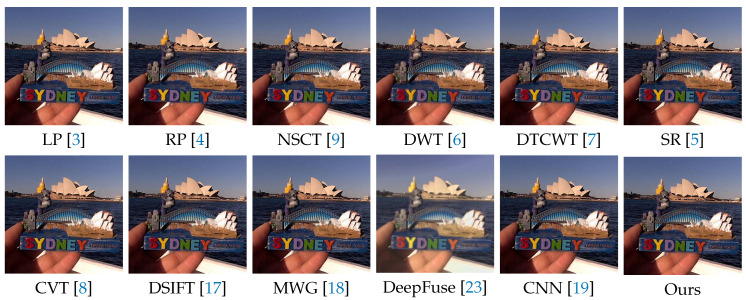
The results of ’Lytro-14’ using various methods.

**Figure 10:**
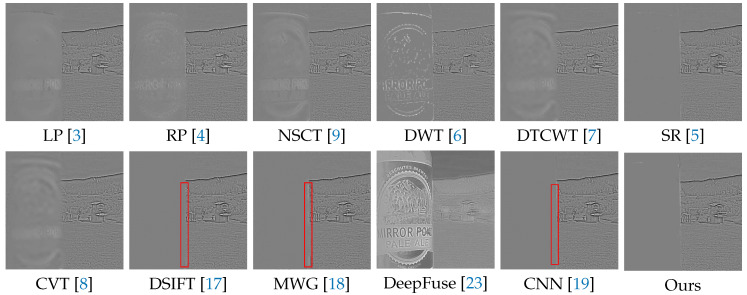
The difference images obtained by subtracting one source image from each fused image (see Figure 7).

**Figure 11:**
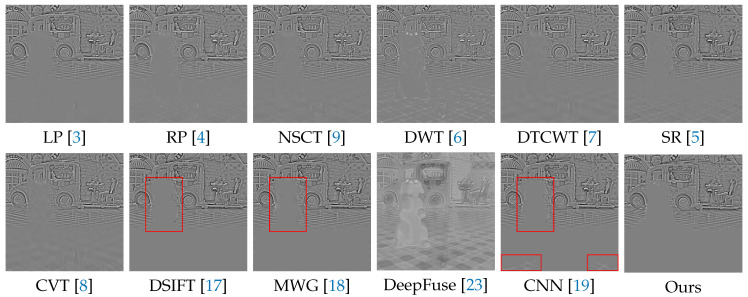
The difference images obtained by subtracting one source image from each fused image (see Figure 8).

**Figure 12:**
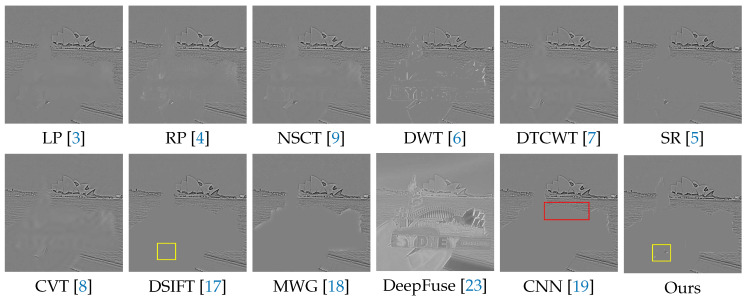
The difference images obtained by subtracting one source image from each fused image (see Figure 9).

We selected 3 pairs of multi-focus images from the Lytro dataset to compare the performance of our method with other methods more intuitively. The fusion results of different methods are visualized in Figure 7, Figure 8 and Figure 9. In addition, for better comparison, we also show the difference image obtained by subtracting one source image from the fused image, as shown in Figure 10, Figure 11 and Figure 12. It is worth noting that if the focus regions are completely detected, there should be no residuals in the corresponding regions on the difference image.

Figure 7 and Figure 10 are visualization results of a beer bottle. We can see that the LP, RP, NSCT, DWT, DTCWT, SR, CVT and DeepFuse methods cannot get desirable fusion results, which is shown as residual information. The DSIFT, MWG and CNN methods can distinguish the focus and defocus regions well, but the boundary between the focus and defocus regions are still slightly curved or unclear that is shown in red box. Our method can get desirable results both inside and at the boundaries of the focus regions.

Figure 8 and Figure 11 show a doll dog on a flat floor. The results of LP, RP, NSCT, DWT, DTCWT, SR, CVT and DeepFuse are not satisfactory, since there are many residuals in the focus regions of the difference image. DSIFT, MWG and CNN have better results, but CNN still has residuals in the lower two red boxes, while our method can completely detect these focus regions. In addition, our method performs better in the near focus boundaries of the toy dog shown in the upper red box.

In Figure 9 and Figure 12, it is a postcard. From the difference images of different methods, we can see that LP, RP, NSCT, DWT, DTCWT, SR, CVT and DeepFuse have more or less residual information in the focus regions such as the hand and postcard, which indicates that they cannot perfectly extract the focus area as a whole. DSIFT, MWG and CNN have good ability to distinguish between the focus and defocus. However, MWG is not clear at the boundary between the focus and defocus regions, which appears as distinct halo artifacts at the edges of the postcard on the difference image. As shown in red box, CNN does not perform well at the curved edges of the upper part of the postcard, where there are rich details. Our method has no error residuals inside the focus regions and is clear at the boundary. It can even detect a defocus region with a small area between fingers which is marked out by the yellow box.

**Figure 13:**
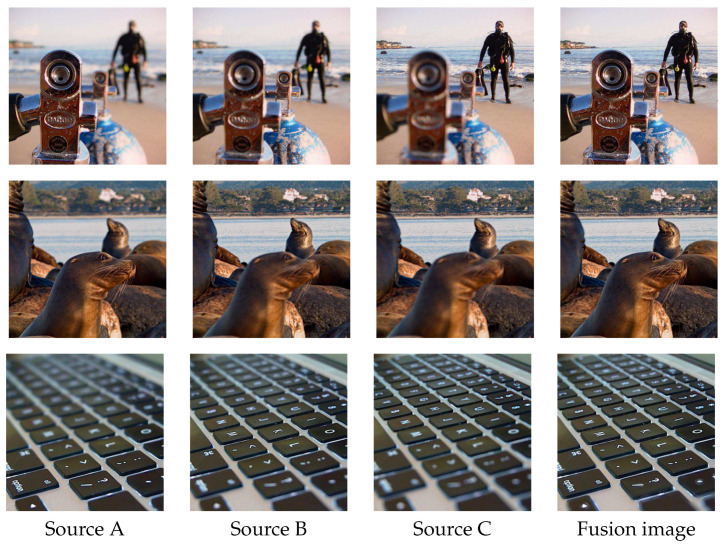
Fusion results of triple source images. Source A, Source B and Source C are images focused on three different distances: near, middle and far. Fusion image is the final fusion result.

We also do experiments on multi-focus image series fusion. Figure 13 shows the visualization results of applying our method to fuse the triple source images one by one in sequence. We can see that the final fused image merges the respective focus regions of the source images well, proving that our solution can be widely applied in practical scenarios.

### 4.6. Quantitative Results

**Figure A1:**
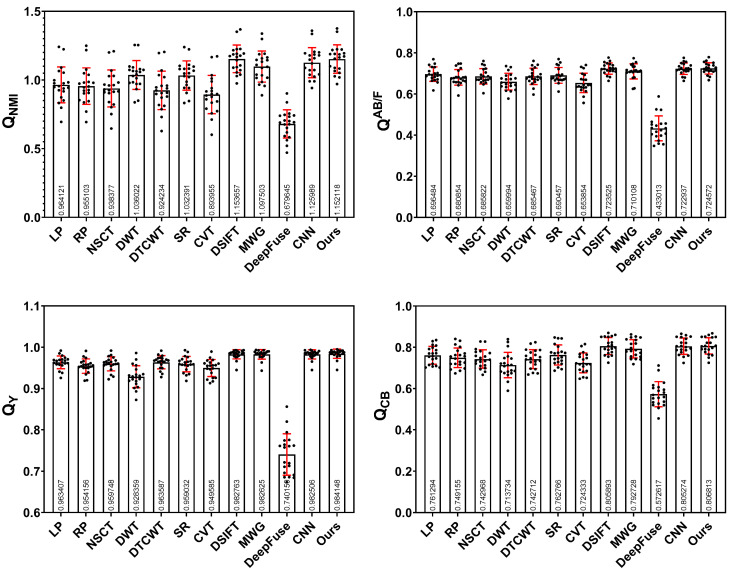
The quantitative results of different methods in the test data (20 pairs of images).

To quantitatively evaluate the performance of the proposed method, we use the four metrics described in Section 4.4 to comprehensively evaluate the fusion results of different methods in the Lytro dataset. The individual data and the means values and standard deviation of the test data for various methods under these metrics are listed in Figure A1 of Appendix A. Table 1 lists the average scores and the highest score is shown in bold. After quantitative comparison, we can see that our method achieves the highest score in three metrics compared to other competitive methods and ranks second in $Q_{NMI}$ behind only DSIFT. The result is consistent with the visual comparison in the previous section. It is still worth noting that our fusion results are obtained directly by the decision map without any post-processing.

Compared with DSIFT, the lower $Q_{NMI}$ means that the less information in the source images is contained in the fused image of our method. However, our method is superior to DSIFT in other metrics, i.e., it has slight advantages in structured information and human perception quality, such as the boundary between the focus and defocus regions, which can be observed in the red box in Figure 10 and Figure 11 and in the yellow box in Figure 12. Moreover, our method has an advantage in terms of time efficiency over DSIFT.

### 4.7. Ablation Study

In this section, we verify the effectiveness of key components in the proposed model through ablation experiments. We will validate in two aspects: the proposed network architecture and the loss function.

#### 4.7.1. Architecture Ablation

**Figure 14:**
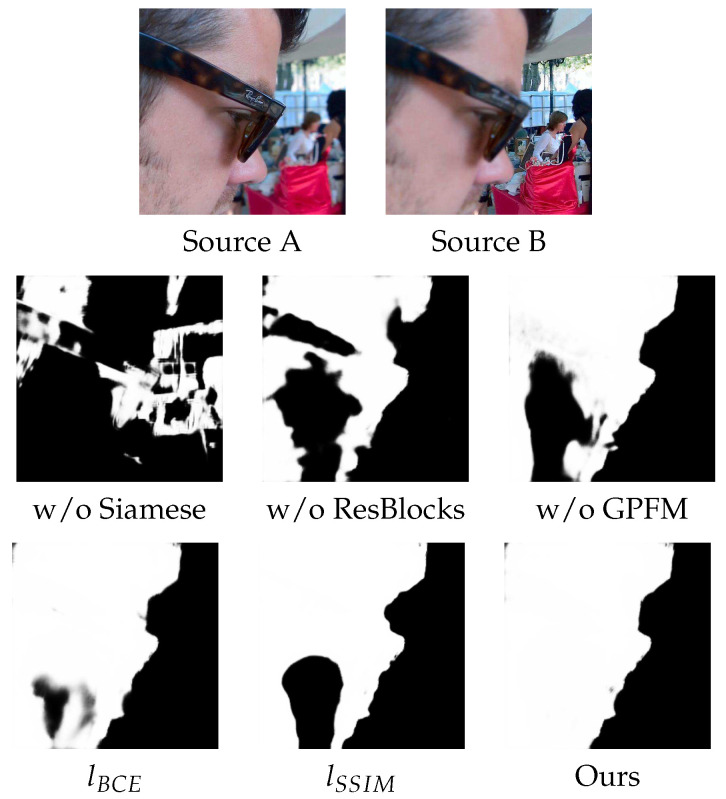
Decision maps generated by different architectures and loss functions. Here, w/o Siamese, w/o ResBlocks and w/o GPFM denote that the Siamese encoder, ResBlocks and the global perception fusion module are removed from the fully implemented network respectively, then train it with the hybrid loss function. $l_{BCE}$ and $l_{SSIM}$ denote that the fully implemented network is trained with the BCE loss and the SSIM loss, respectively.

**Figure A2:**
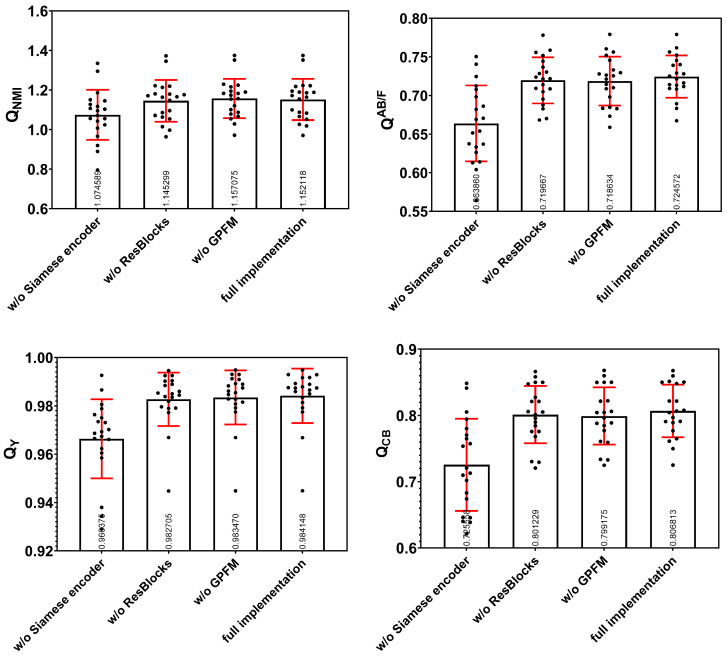
The quantitative results of different network architectures in the test data (20 pairs of images).

As described in Section 3.2, there are three key parts of our proposed architecture: (1) using a Siamese-based encoder to accept dual inputs; (2) Resblocks are used in the encoder and decoder; (3) Global perception fusion module is introduced into the bridge stage of the network. To verify the effectiveness of these three components, we removed them separately from the full implementation and reported their results on quantitative experiments. In particular, we concatenate the two images at the input stage to replace the Siamese encoder. The individual data and the means values and standard deviation of the test data for various network architectures are listed in Figure A2 of Appendix A and the average scores of this architecture ablation are shown in Table 2. It illustrates the effectiveness of these three components, and our full implementation achieves the best performance. Specifically, the lack of Siamese encoder makes each metric value drop significantly, which indicates that the Siamese encoder is the most important module. Moreover, we can observe that the biggest change brought by Siamese encoder is in the image visual quality. Similarly, the absence of the ResBlocks results in a decrease in each metric value, which demonstrates the effectiveness of the ResBlocks. Lacking GPFM results in a slight increase in $Q_{NMI}$, but a significant decrease in the $Q^{AB/F}$ and $Q_{CB}$, indicating that the GPFM module reduces the information of the source image contained in the fusion results but focuses on the edge information and overall visual performance in the output. To illustrate the effect of these components more intuitively, Figure 14 shows the decision maps generated by different network architectures. It is obvious that our complete architecture achieves desirable qualitative results.

#### 4.7.2. Loss Ablation

**Figure A3:**
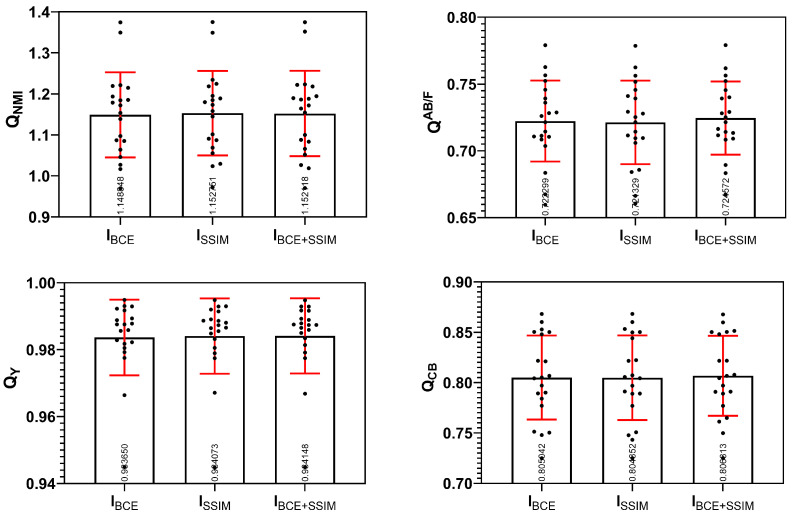
The quantitative results of different loss functions in the test data (20 pairs of images).

To verify the effectiveness of our proposed hybrid loss combining the BCE loss and the SSIM loss, we conduct comparative experiments on models trained with only the BCE loss, only the SSIM loss and the hybrid loss. The individual data and the means values and standard deviation of the test data for various loss functions are listed in Figure A3 of Appendix A. The average quantitative results in Table 3 illustrate that the proposed hybrid loss can effectively improve the performance of our network. Specifically, we can observe that the lack of the SSIM loss in the second row of this table makes the value of each metric drop, i.e., the SSIM loss is very important. As can be seen in line 3, lacking the BCE loss results in a slight increase in $Q_{NMI}$ and a decrease in other metrics, indicating that the BCE loss focuses on the overall structure and visual quality of the image while reducing the mutual information between the source images and the fused image. Comparing the value of $Q_{Y}$, we can see that the lack of the SSIM loss brings a greater decrease than the lack of the BCE loss, indicating that the SSIM loss does pay attention to structural information. Figure 14 also shows the decision maps generated by our network model trained with different loss functions. As we can see, the proposed hybrid loss function achieves the best qualitative results.

### 4.8. Computational Efficiency

To evaluate the computational efficiency, we list the average running time of various algorithms on the Lytro dataset in Table 4. Obviously, the time consumption of our method is only 0.06 s, which is faster than other algorithms. It shows that our method can have good usage in practice.

## 5. Conclusions

In this paper, we propose a novel U-shape network with a Siamese structured encoder for the multi-focus image fusion task. The U-shape Siamese network can preserve multi-level features from two source image to enhance the generated decision map. ResBlocks are introduced in the network to increase the receptive field, which helps to better perceive the focus characteristics of the image. In addition, a global perception fusion module based on spatial pyramid pooling is added to obtain context information. A hybrid objective combining BCE loss and SSIM loss is used to train our model on a multi-focus image dataset, which is synthesized on the VOC 2012 natural image dataset. Experimental results show that our proposed method achieves the start-of-the-art performance both in visual perception and quantitative evaluation.

## Figures and Tables

**Table 1:** Average scores of various methods on the four metrics.

Metric	$Q_{NMI}$	$Q^{AB/F}$	$Q_{Y}$	$Q_{CB}$
LP [3]	0.964121	0.696484	0.963407	0.761294
RP [4]	0.955103	0.680854	0.954156	0.749155
NSCT [9]	0.938377	0.685822	0.959748	0.742968
DWT [6]	1.036022	0.659993	0.928359	0.713734
DTCWT [7]	0.924234	0.685467	0.963587	0.742712
SR [5]	1.032391	0.690457	0.959032	0.762765
CVT [8]	0.893955	0.653854	0.949585	0.724333
DSIFT [17]	**1.153657**	0.723525	0.982763	0.805893
MWG [18]	1.097503	0.710108	0.982625	0.792728
DeepFuse [23]	0.679645	0.433013	0.740159	0.572617
CNN [19]	1.125989	0.722936	0.982505	0.805273
Ours	1.152118	**0.724572**	**0.984148**	**0.806813**

**Table 2:** The effectiveness of our different key components in the proposed model.

Metric	$Q_{NMI}$	$Q^{AB/F}$	$Q_{Y}$	$Q_{CB}$
w/o Siamese encoder	1.074588	0.663860	0.966375	0.725558
w/o ResBlocks	1.145299	0.719667	0.982705	0.801229
w/o GPFM	**1.157074**	0.718634	0.983470	0.799175
full implementation	1.152118	**0.724572**	**0.984148**	**0.806813**

**Table 3:** The effectiveness of our hybrid loss function.

Metric	$Q_{NMI}$	$Q^{AB/F}$	$Q_{Y}$	$Q_{CB}$
$l_{BCE}$	1.148848	0.722299	0.983650	0.805042
$l_{SSIM}$	**1.152751**	0.721329	0.984072	0.804851
$l_{BCE+SSIM}$	1.152118	**0.724572**	**0.984148**	**0.806813**

**Table 4:** Average running time of various methods. (Unit:Seconds).

Method	LP [3]	RP [4]	NSCT [9]	DWT [6]	DTCWT [7]	SR [5]
Time	14.60	14.59	25.29	14.70	15.20	226.82
Method	CVT [8]	DSIFT [17]	MWG [18]	DeepFuse [23]	CNN [19]	Ours
Time	17.29	30.92	20.42	0.74	142.97	**0.06**

## Appendix A

The four metric values of each method in each image, namely all the data, are shown in Figure A1–Figure A3.
